# Orthology Detection Combining Clustering and Synteny for Very Large Datasets

**DOI:** 10.1371/journal.pone.0105015

**Published:** 2014-08-19

**Authors:** Marcus Lechner, Maribel Hernandez-Rosales, Daniel Doerr, Nicolas Wieseke, Annelyse Thévenin, Jens Stoye, Roland K. Hartmann, Sonja J. Prohaska, Peter F. Stadler

**Affiliations:** 1 Institut für Pharmazeutische Chemie, Philipps-Universität Marburg, Marburg, Germany; 2 Bioinformatics Group, Department of Computer Science, Universität Leipzig, Leipzig, Germany; 3 Interdisciplinary Center for Bioinformatics, Universität Leipzig, Leipzig, Germany; 4 Max Planck Institute for Mathematics in the Sciences, Leipzig, Germany; 5 Departamento de Ciência da Computação, Instituto de Ciências Exatas, Universidade de Brasília, Brasília, Brasil; 6 Genome Informatics, Faculty of Technology, Bielefeld University, Bielefeld, Germany; 7 Institute for Bioinformatics, Center for Biotechnology, Bielefeld University, Bielefeld, Germany; 8 Faculty of Mathematics and Computer Science University of Leipzig, Leipzig, Germany; 9 Computational EvoDevo Group, Department of Computer Science, Universität Leipzig, Leipzig, Germany; 10 Institute for Theoretical Chemistry, University of Vienna, Vienna, Austria; 11 Center for non-coding RNA in Technology and Health, University of Copenhagen, Frederiksberg, Denmark; 12 The Santa Fe Institute, Santa Fe, New Mexico, United States of America; 13 RNomics Group, Fraunhofer Institut for Cell Therapy and Immunology, Leipzig, Germany; Hellas, Greece

## Abstract

The elucidation of orthology relationships is an important step both in gene function prediction as well as towards understanding patterns of sequence evolution. Orthology assignments are usually derived directly from sequence similarities for large data because more exact approaches exhibit too high computational costs. Here we present PoFF, an extension for the standalone tool Proteinortho, which enhances orthology detection by combining clustering, sequence similarity, and synteny. In the course of this work, FFAdj-MCS, a heuristic that assesses pairwise gene order using adjacencies (a similarity measure related to the breakpoint distance) was adapted to support multiple linear chromosomes and extended to detect duplicated regions. PoFF largely reduces the number of false positives and enables more fine-grained predictions than purely similarity-based approaches. The extension maintains the low memory requirements and the efficient concurrency options of its basis Proteinortho, making the software applicable to very large datasets.

## Introduction

Detailed knowledge on the history of large gene families is crucial to the understanding of their patterns of sequence evolution and their functional interpretation. Throughout this contribution we use the term “gene” to denote any genomic feature that can be represented as a sequence interval. No further functional or structural properties are implied. An important step towards this goal is the elucidation of orthology relationships. Two genes are orthologs if they arose via a speciation event from their last common ancestor in the gene tree. In contrast, paralogs originate from a gene duplication event [1], [2]. The definition of orthology implies that an event-annotated gene tree is available, and thus a gene tree and its reconciliation with the underlying species tree must be known to determine with certainty which pairs of genes are orthologs. Since ancestral states are in general experimentally inaccessible, the orthology relation, just like the gene phylogeny, has to be inferred from extant sequence data.

A large class of orthology detection tools therefore attempts to explicitly infer gene phylogenies and their reconciliation with species trees, e.g. Orthology analysis using MCMC [3], MultiMSOAR [4], LOFT [5], Ensembl Compara [6], and Synergy [7]. Although this tree-based approach is often considered the most accurate, it suffers from high computational costs and is hence limited in practice to a moderate number of species and genes. Moreover, all practical issues that hamper phylogenetic inference (e.g. variability of evolutionary rate, mistaken homology, homoplasy, and horizontal gene transfer) limit the accuracy of both the gene and the species trees.

The second class of algorithms bypasses the construction of gene and species trees by directly deriving orthology assignments from similarity data. Approaches of this type are COG [8], OrthoMCL [9], [10], OMA [11], [12], InParanoid [13], eggNOG [14], HomoloGene [15], Roundup 2.0 [16], or EGM2 [17]. Since orthology is not a transitive relation, the problem of orthology detection is fundamentally different from clustering or partitioning of the input gene set. In particular, a set *A* of genes can be orthologous to another gene 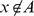 but the genes within *A* are not necessarily orthologous to each other. In this case, the genes in *A* are called co-orthologs to gene *x* [18]. A common feature of most of the methods mentioned above is that they do not produce an estimate for the pairwise orthology relations but return orthologous groups containing genes which are mutually orthologous to the greatest extent but also comprise co-orthologous genes. We refer to these groups as orthologous groups in the following. In addition to OMA and Proteinortho [19], only Synergy, EGM2, and InParanoid attempt to resolve the orthology relation at the level of gene pairs. The latter two tools can only be used for the analysis of two species at a time, while Synergy is not available as standalone tool and therefore cannot be applied to arbitrary user-defined datasets. The use of these tools is limited to the species offered through the databases published by their authors.

The orthology relation can be represented as a graph on the set of genes. It forms a cograph rather than a partition [20]. Clustering approaches identify dense subgraphs of these cographs and hence introduce false-positive edges corresponding to recent paralogs. On the other hand, ancient paralogs are often separated into different groups of co-orthologs. Despite this theoretical shortcoming, cluster-based methods have consistently been reported to yield very good results [21]–[23]. Since they are much faster than tree-based algorithms, they can be applied to very large datasets.

The clustering method and, in many cases, user-defined parameters determine the granularity of the orthologous groups and thus the tolerance to false positive orthology assignments. Some methods are very inclusive [5], but the aim typically is to remove as many paralogs as possible to approach a one-to-one orthology relation. These simple relationships are especially useful for phylogenetic analysis and for exact functional predictions. Phylogenomic studies typically employ pipelines such as HaMStR [24] to restrict the data to one-to-one orthologs. When the phylogenetic range of interest includes duplication events however, such approaches are bound to fail [25].

Here we focus on an intermediate balance. Our main aim is to avoid false positive orthology assignments within the phylogenetic range of the reported orthologous groups, while we tolerate recent in-paralogs (speciation preceding duplication) as unavoidable contamination. Clustering approaches for orthology detection are usually based on the “best match method”, which attempts to find orthologs as the sequence in another genome that is most similar to the query. It often fails in the presence of paralogs with comparable similarity to the query. Best match approaches are nevertheless routinely used to gain insight into relationships of genes among phylogenetically very diverse organisms. These approaches are used in particular for gene annotation in newly sequenced genomes for which a well studied close relative is lacking. However, the large number of sequencing projects of the last decade have largely reduced the gaping holes in phylogenetic coverage and most large-scale comparative studies nowadays focus on closely related species or even strains [26], [27]. As a result, the evolutionary distances within a phylogeny of interest are often rather small, hence additional information to resolve evolutionary relationships between genes can be obtained from genomic context. Furthermore synteny, i.e., the conservation of gene order (also referred to as gene context) provides information independent of sequence similarity, which can help to sort paralogs. Both Synergy and EGM2 incorporate synteny information to compute orthology relations. The Synergy algorithm achieves high accuracy due to the fact that it reconstructs gene family trees [28]. EGM2 considers synteny by identifying similar genomic regions to detect orthologs. However, this tool is not suitable for large datasets due to its restriction to only two genomes at a time. Genes with a common ancestry that are functionally linked with each other frequently show a conservation in local gene order over long evolutionary distances [29], [30]. Thus, synteny is frequently used to disentangle complex duplication histories, see e.g. [31] and references therein. The intricacies of conserved synteny and positional orthology have been reviewed recently [32].

The computational prediction of syntenic regions usually relies on the detection of genomic neighborhoods that are conserved between genomes of related species. Proximity relations among genes, such as adjacencies [33] (two genes encoded adjacent to each other in several genomes), generalized adjacencies [34] or conserved intervals [35], are used to assess genomic neighborhoods. Typical methods for the detection of syntenic regions utilize gene family information, similarity scores or conserved distances to establish putative homologies and then apply chaining or clustering algorithms. When paralogous genes are considered, the underlying computational problems become prohibitive because many alternative synteny assignments are possible. Exact algorithms are therefore slow and limited to small datasets. In fact, the problem of computing the syntenic distance between two genomes is NP-hard [36], [37]. Efficient heuristics are therefore employed to deal with large datasets.

If gene family information is available, popular synteny tools such as i-ADHoRe 3.0 [38] and MCScanX [39] can efficiently detect homologous regions even in large-scale analyses. Otherwise, using local alignments of sequences, tools such as CYNTENATOR [40] and DAGchainer [41] allow for detection of syntenic regions based on pairwise similarity scores of sequence intervals. The heuristic method FFAdj-MCS [42] has proven to be a good compromise in terms of both, speed and accuracy, as it takes a different approach by calculating a matching whose objective function maximizes towards a balance between adjacencies and similarity scores of genes.

In this contribution we describe PoFF, an extension of Proteinortho [19], to include synteny information in a systematic way. More precisely, a pair of genes (*A*1, *A*2) in genome *A* is considered syntenous with another pair of genes (*B*1, *B*2) in genome *B*, if both *A*1, *B*1 and *A*2, *B*2 are potential orthologs (as determined by sequence similarity), and both (*A*1, *A*2) as well as (*B*1, *B*2) are adjacent gene pairs on their corresponding chrosomosomal locations. In case other genes are located between (*A*1, *A*2) or (*B*1, *B*2), these must not be orthologous to any other genes in genomes *A* or *B*. Proteinortho applies an adaptive best match method together with spectral clustering to define (co-)orthologs. Its performance in terms of accuracy has been shown to be comparable to other clustering-based methods. At the same time it has modest requirements in terms of memory and computation time and is thus suitable for very large datasets. Complementing the evaluation of pairwise sequence similarities, we incorporate here the efficient heuristic algorithm FFAdj-MCS that computes ortholog assignments by maximizing the above synteny measure between pairs of genomes. Following a recent suggestion [43], true orthologs among multiple candidates were defined as those that retained their original genomic context. In the course of this work, we adapted FFAdj-MCS to include multiple linear chromosomes within single organisms and extended it for the detection of duplicated genes and large duplicated genomic regions. We note that the algorithm may also be applied to circular chromosomes at the expense of losing synteny information for at most two pairs of genes at the very ends of the linearized representation. This minor shortcoming should have no or only a vanishingly low effect in the overall process of orthology assignment.

**Figure 1:**
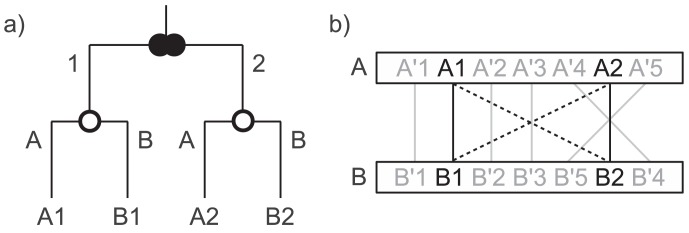
Synteny-enhanced orthology prediction. Four genes (*A*1, *A*2, *B*1, *B*2) in two species (*A* and *B*). a) The gene tree with a duplication (filled double circle) and a speciation event (empty circle). b) Gene order in the genomic context of both genes. Genes *A'x* and *B'x* are orthologous to each other. Lines depict suggested partners based on sequence similarity of which the dashed were neglected by the gene order algorithm.

Figure 1 illustrates the idea of the synteny-enhanced version of Proteinortho. In this example, four genes (*A*1, *A*2, *B*1, *B*2) in two species (*A* and *B*) are considered. The gene tree in Figure 1a shows a duplication preceding a speciation event. *A*1 and *B*1 as well as *A*2 and *B*2 are orthologous to each other as they derived from a common ancestor by speciation. Given sufficient similarity, however, all four genes would be reported as an orthologous group using regular sequence similarity-based approaches. The gene order depicted in Figure 1b allows one to distinguish the genes 1 and 2 from each other. The combined approach therefore predicts the two distinct orthologous groups {*A*1, *B*1} and {*A*2, *B*2} and thus avoids false positive orthology assignments.

We argue that the level of granularity achieved in this way is more useful in most cases than an arbitrary separation of groups solely based on sequence similarity scores which tend to lack significance when sequences are closely related. The same holds compared to inclusive strategies which hardly discriminate subgroups. Assuming that numerous extant genes have derived from a limited set of common ancestors by a series of duplication events, inclusive strategies will include entire gene families, and hence lead to very large groups with a significant amount of actually non-orthologous genes. An emphasis on including all pairwise orthology relations when reporting orthologous groups thus seems to be of little use.

We evaluated PoFF using several sets of simulated protein-coding genes. Each set was derived from event-annotated gene trees. Thus, for each pair of genes, the true relationship regarding orthology is unambiguously defined and used to validate the predictions. Our results reveal a significant improvement with respect to true negative and false positive predictions at the expense of only a marginal decrease of the true positive rate.

## Materials and Methods

### Conceptual Outline

Our starting point for orthology detection is a directed graph 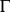 whose vertices are all the genes of all input genomes. A directed edge 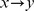 is introduced if (i) *x* and *y* are taken from two different genomes (*A* and *B*) and (ii) the similarity *s*(*x*, *y*) is not much smaller than the gene *z* in *B* that is most similar to *x*, i.e., if 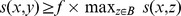 for some stringency parameter *f*≤1. Since any true ortholog of 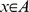 in genome *B* should be among the most similar sequences that can be found in *B*, 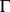 should have few false negatives (i.e. missing true edges) as long as the stringency is not set to a value that is too restrictive. The idea is, therefore, to remove edges from the graph 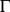 that are likely false positives. Since orthology is a symmetric relation, we only retain edges 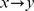 if 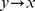 is also contained in 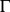.

Synteny information determined by FFAdj-MCS provides an additional filter for the edge set of 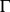. By construction, the subgraph 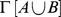 induced by the genes in *A* and *B* is bipartite. Synteny is modeled as the relative order of edges along both genomes. Synteny as a filter reduces the edge set of 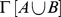 to a matching that maximizes a trade-off between the total number of edges and the number of conserved adjacencies. Among similar paralogs, this strategy favors the one with the best-conserved local gene order as representative of the orthologous group. In the final step, a clustering algorithm [19] is employed to extract groups of co-orthologs from 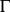, which contains all subgraphs 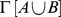 for all pairs of genomes.

### Implementation

**Figure 2:**
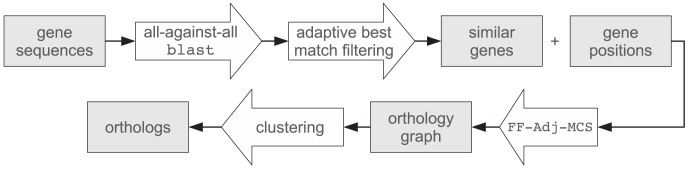
Workflow of PoFF. Similar gene sequences are determined by an all-against-all blast search. Top reciprocal matches are ordered by their positions in the respective genomes. The FFAdj-MCS algorithm is applied to determine the maximum matching with respect to sequence similarity and gene order. As a result the orthology graph 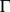 only contains the remaining edges from pairwise comparisons. Finally, orthologous groups are extracted by clustering.

Proteinortho uses the blast bit score to determine potential homologs in another species and to measure sequence similarity. The definition of the edge set above makes it possible to construct 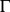 directly from pairwise comparisons. Thus, this initial state can be trivially parallelized and does not require the storage of genome-wide blast comparison data in memory. As the FFAdj-MCS algorithm applies to pairs of genomes *A* and *B*, it can be added to the workflow without breaking these advantageous properties. The algorithm requires information on gene order and pairwise gene similarity for two genomes and determines a matching that maximizes a weighted sum of edge weights and weights of conserved adjacencies. To this end FFAdj-MCS matches genes in regions with conserved gene order that locally maximize the objective of FF-Adjacencies [42]. These regions are called maximum common substrings (MCSs). Since the blast scores *s*(*x*, *y*) are not symmetric, they are symmetrized (taking the average of both scores) for use in FFAdj-MCS. The combination PoFF of Proteinortho and FFAdj-MCS yields, for each pair of genomes, a pruned set of edges that is highly enriched in true orthologous pairs. The workflow of our extension is illustrated in Figure 2.

We added three extensions to the FFAdj-MCS program as presented in [42]. Firstly, it was adapted to allow for more than one chromosome per genome. Secondly, the detection of duplicated genes and large duplicated regions was implemented: The heuristic was adapted to repeat a user-defined number of complete matchings, where edges selected by preceding matchings are removed before each subsequent matching. Thirdly, FFAdj-MCS allows to filter the size of MCSs obtained from subsequent matchings by means of a user-defined minimal size 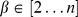 that defines the minimum number of gene pairs in each MCS.

Finally, we relaxed the criteria for very similar neighboring genes: If two adjacent genes *x* and *y* in *A* both have their best alignment to the same gene 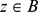, we include both edges {*x*, *y*} and {*y*, *z*} in 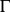, since *x* and *y* are likely in-paralogs and a decision for one of the two edges based on a small score difference is not reasonable from a biological perspective. Even though this makes PoFF more inclusive, we argue that this behavior is more reasonable because such in-paralogs can be quite easily detected in a post-processing step if required for a particular application.

PoFF has several parameters that can be set by the user – in particular score thresholds and coverage requirements of the blast searches. We used the default settings throughout. The stringency parameter *f* defines the fraction of the bit score of the best blast hit that must be reached by an alternative candidate ortholog. Proteinortho's default value, *f* = 0.95, has been shown to work also in conjunction with the synteny filter. The FFAdj-MCS algorithm provides an adjustable parameter 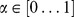 that controls the relative importance of edge weights and the weights of adjacencies. Benchmarking PoFF did not reveal a strong dependency of the results on this parameter, likely because nucleotide sequences and order of genes evolve in parallel and with comparable speeds. We therefore used the default value 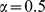 throughout. By default, we perform one matching iteration with 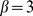 to cover the detection of large duplicated regions. If multiple copies of a region are expected in a dataset, this number of iterative matchings can be increased further. Practical experience with Proteinortho also led to the decision to increase the default E-value threshold from 10^−10^ to 10^−5^ in order to improve coverage of less conserved orthologs.

### Benchmarking

Since implementations of competing tools that generate fully resolved orthology relations are not publicly available, we cannot employ the usual evaluation strategy of comparing all tools on series of benchmarking datasets of our choice. Instead we apply both Proteinortho and PoFF to several reference datasets that either comprise simulated data for which the underlying gene trees are known, or real data which defines orthologous groups and/or pairwise orthologous relationships by extensive analysis, often including manual curation. Results are then compared to the published performance of alternative tools.

For Proteinortho and PoFF we used standard parameters, including an E-value threshold of 10^−5^. However, the more recent blastp+ software [44] instead of the original blastp implementation [45] was applied to find the initial matches. This can be set by a parameter in Proteinortho.

The generation of simulated data is described below. As some of these sets were sufficiently small, we also applied OrthoMCL, OMA and InParanoid in order to evaluate the results. Again, standard parameters were used, including an E-value threshold of 10^−5^ for OrthoMCL. Real life data was taken from various sources also described hereafter. The YGOB dataset [46], was used in a previous study to evaluate the Synergy approach [7]. Hence, we took the opportunity to include the available results to the benchmark.

#### Simulated data

In the absence of extensive gold standard datasets comprising sequence and synteny data as well as the underlying gene trees that could be used for benchmarking our orthology prediction method, we simulated sequence evolution and genomic rearrangements on a single chromosome for three example datasets comprising 50, 80 and 100 gene families (proteins) in 20 species (named hereafter *F*50, *F*80*d* and *F*100, respectively). All test sets feature duplications of both individual genes and gene clusters. The set *F*80*d* in addition includes whole genome duplications. Table 1 gives a closer look to the composition of all three datasets as well as to their average breakpoint distances determined by PoFF. The simulation pipeline is available in the Online Supplemental Material.

**Table 1:** Composition of simulated datasets.

Dataset	Families	Proteins	ø Family size	ø Breakpoint distance
*F*50	50	8,363	167 proteins	13
*F*80*d*	80	15,296	191 proteins	19
*F*100	100	27,258	273 proteins	14

The simulated datasets differ by the number of gene families present in the species as well as by the size of these families. The larger the families the more diversity among the set of species can be considered. Set *F*80*d* additionally comprises whole genome duplications.

Species trees were simulated according to the *Age Model* [47]. These trees are balanced and edge lengths are normalized so that the total length of the path from the root to each leaf is 1. For each species tree *S*, we then simulated gene trees using the following rules:

1. The root of *S* contains an ordered list of ancestral genes one for each gene family. The number of families is a user-defined parameter.
2. *S* is traversed in a depth first order. All changes to the genome are simulated independently for each edge of *S* with constant rates.
3. At each internal node of *S*, the ordered gene list received from its parental edge is copied without change to both offspring edges.
4. Along each edge of *S* a number of events is sampled from a Poisson Process 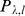, where the parameter 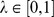 is the probability of the event to happen and *l* is the branch length. The process may generate none, one, or several events of the following types: gene duplication, cluster duplication, genome duplication, and gene loss. Here we used the parameters 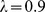 for gene duplication, 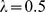 for cluster duplication, 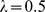 for gene loss. For the dataset *F*80*d* we consider genome duplications with 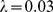 instead.
5. A special rule applies to recently duplicated genes to account for the deletion of redundant gene copies before they can be stabilized by sufficient functional divergence or subfunctionalization [48], [49]. We model this by a probability of 0.3.
6. To obtain an order of the generated genes, rearrangements are carried out for each edge of *S* using translocation and inversion operations on the ordered list of genes that “survived” until the next speciation. Rearrangements are picked randomly and the number of inversion operations is chosen uniformly proportional to the branch length [50].

**Figure 3:**
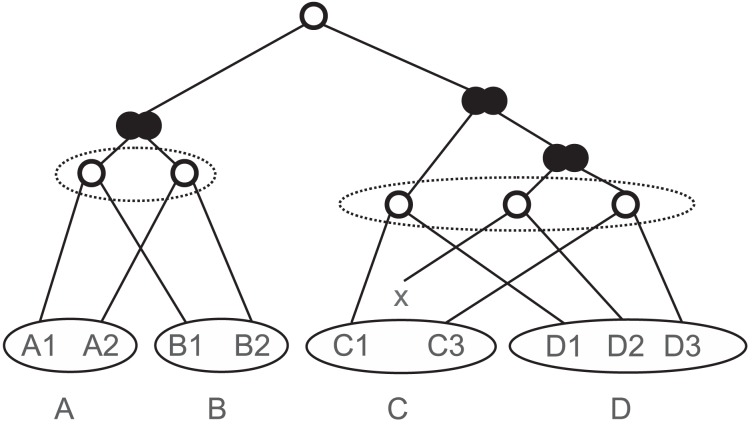
A reconciled tree for gene families. The gene tree is embedded in the species tree. Internal nodes represent either gene duplication (filled double circle) or speciation events (empty circles). Gene loss is depicted by ×.

The result of this simulation is a gene tree *G_i_* for each family *i* together with a true reconciliation map to the species tree *S*. All gene lineages terminating in a deletion event are pruned from the gene tree so that we retain a gene tree *G_i_* in which only extant genes appear as its leaves. The known reconciliation furthermore provides us with a labeling of the internal nodes of *G_i_* with *duplication* or *speciation* events, see Figure 3 for an example. This in turn determines the true orthology relation for all genes received in the leaves of *S*. In addition, the gene orders within their respective genomes are obtained. The simulations were performed using a simulation environment for large gene families [51].

Since large-scale orthology analysis are usually performed for protein sequences, we use indel-Seq-Gen [52] to generate simulated amino acid (aa) sequences for the gene trees *G_i_*. For each gene family a random seed sequence is initiated with a length between 100 and 1,000 aa. Then, to define the offspring genes, indel-Seq-Gen introduces substitutions according to PAM substitution matrix and insertions and deletions with a Zipfian probability distribution [53] with maximal length between 1% to 10% of the sequence length. For the gene trees a branch scale factor of 0.5 was used. This is the frequency of a single amino acid to be substituted. Hence, approximately half of the amino acids are changed during the simulation on the path from the root to the leaf.

We remark that the Artificial Life Framework (ALF) [54] for simulating sequence evolution could in principle have been used for simulating test data. However, in its current version, this tool does not support genome-wide duplications and selective loss of recently duplicated genes. We therefore opted to construct our own simulation framework.

#### Real life data

COG: We used proteome data from the COG-database, which provides manually curated orthology relations (ftp://ftp.ncbi.nih.gov/pub/COG/COG/, 2009/10/15), for the following set of 16 species covering three bacterial groups: *Bacillus halodurans*, *Bacillus subtilis*, *Lactococcus lactis*, *Listeria innocua*, *Streptococcus pneumoniae* TIGR4, *Streptococcus pyogenes* M1 GAS from the Gram-positive bacilli class, *Buchnera sp.* APS, *Escherichia coli* K12, *Pasteurella multocida*, *Salmonella typhimurium* LT2, *Vibrio cholerae*, *Yersinia pestis* from the gamma proteobacteria class and *Brucella melitensis*, *Caulobacter vibrioides*, *Mesorhizobium loti*, *Rickettsia prowazekii* from the alpha proteobacteria class. According to PoFF, the average breakpoint distance of this set is 642.

To obtain the gene orders we retrieved the genomes from the NCBI-database (ftp://ftp.ncbi.nih.gov/genomes/Bacteria/, 2012/12/13, see supplement). When several strains were available, we picked the one with the smallest uid as they represent the older genomes preferentially included in secondary databases. All genes were then located in the genomes using tblastn+ with an E-value threshold of 10^−8^. The best match was considered to be the gene of interest. A small minority of genes (98 out of 53,264) could not be located unambiguously and was thus removed from the dataset.

As we used an extract of the COG-database (16 out of 66 species), only COG-groups covering at least eight proteins within the set of the chosen 16 species were considered to estimate the orthology matrix as described below (see Evaluation). Otherwise, their classification might have been based on species not in the dataset used here, which would make a comparison of approaches unreasonable.

OrthoBench: We also used the reference annotation OrthoBench [23]. Manually curated orthologous groups were downloaded from http://eggnog.embl.de/orthobench/ at 2013/01/05. The set comprises 12 metazoan proteomes and is based on the Ensembl *v*60 genome annotation [55] which was downloaded from ftp://ftp.ensembl.org/pub/release-60/ at 2013/01/11. According to PoFF, the average breakpoint distance of this set is 5,433. 124 out of 1,692 proteins stated in OrthoBench could not be located in the *v*60 set and were excluded from the analysis.

YGOB: From this dataset we obtained orthology assignments of five ascomycete fungi *Ashbya gossypii*, *Saccharomyces cerevisiae*, *Candida glabrata*, *Kluyveromyces lactis*, and *Kluyveromyces waltii* that have been used in the evaluation of Synergy in the original study [7]. According to PoFF, the average breakpoint distance of this set is 2, 697. The data provided by the authors included pairwise blast results with an E-value threshold of 10^−5^, which we directly used in our analysis, omitting the blast step. In this way, the initial blast data on which Synergy, Proteinortho, and PoFF operated was assured to be identical. We then compared orthologs predicted by the three approaches to orthologs from the YGOB dataset (v1, 2005) [46]. We excluded genes from the predictions that were not contained in the YGOB dataset (6, 218, 6, 076 and 6, 817 out of 23, 134 for Proteinortho, PoFF and Synergy, respectively). In this way, we avoid to bias our evaluation with data that is not present in the reference dataset.

#### Evaluation

For each gene family/orthologous group in the reference sets, we compared the pairwise orthologous relationships between its members to the predictions, counting true positives (*tp*), false positives (*fp*), true negatives (*tn*) and false negatives (*fn*) as well as the number of orthology relations between reference groups. These data were then used for statistics as follows:
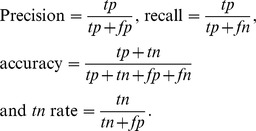



For evaluation of PoFF and Proteinortho, we used the orthology graph returned in addition to orthologous groups which contains information on pairwise orthology relations. OMA returns this graph equivalently in the PairwiseOrthologs output. InParanoid was applied to all pairs of species successively. After merging the results, this resulted in pairwise orthology relations for the whole dataset as well. OrthoMCL on the other hand, does not return the orthology graph directly. We extracted the information on pairwise orthology relations from the MCL clustering output file. Connected components in there are used by OrthoMCL to determine orthologous groups, making this output file similar to the orthology graph returned by PoFF/Proteinortho. We note however, that the file was not meant to be used as orthology graph. Given the mode of calculation applied by OrthoMCL, it contains numerous orthology relations for paralogs of the same species which cannot occur using PoFF/Proteinortho. For Synergy and YGOB, pairwise orthology relations were present. For COG and OrthoBench, however, only data on orthologous groups was provided. The pairwise orthology relations had to be estimated. We did this by assuming each protein of an orthologous group to be orthologous to each other protein in the same group, except when both proteins belong to the same species. We emphasize that this strategy strictly overestimates the number of orthologous relationships in the dataset. Nonetheless, this method makes it possible to compare the results on a pairwise level.

The simulated data also provides gene trees. These were used to acquire pairwise orthology relations. Two genes of a simulated gene family are orthologous to each other, if and only if their most recent common ancestor event was a speciation.

## Results and Discussion

In order to estimate how PoFF performs with respect to closely related species and compared to the original Proteinortho implementation, we simulated and subsequently evaluated three datasets (*F*50, *F*80*d*, *F*100), for which the gene histories and hence the true orthology relations are defined. The datasets differ in number and size of gene families, thus representing increasing levels of diversity among closely related species. The results are summarized in Table 2. Proteinortho already performs very efficiently. However, as the number of paralogs with similar sequences increases, the basic algorithm becomes less effective in precisely predicting the correct orthology relations within these gene families, a trend that exacerbates with increasing size of gene families. The use of the synteny information provided by the FFAdj-MCS algorithm efficiently counteracts this tendency and substantially improves the precision. Other performance statistics as well as the runtime remain nearly unchanged, which indicates a significant advantage of PoFF over the original Proteinortho tool.

**Table 2:** Comparison using simulated data.

Dataset	Method	Precision	Recall	Accuracy	*tn* rate	Runtime
F50	OrthoMCL	3.06%	7.26%	86.18%	89.71%	7 h, 22 min
	OMA	38.64%	9.62%	95.49%	99.32%	1 day, 14 h
	InParanoid	98.01%	5.02%	95.94%	99.99%	2 days, 2 h
	Proteinortho	80.63%	23.11%	97.62%	99.83%	0 h, 36 min
	PoFF	96.15%	24.18%	97.53%	99.96%	0 h, 36 min
F80d	OrthoMCL	0.92%	0.88%	87.44%	93.43%	15 h, 46 min
	OMA	43.97%	5.25%	93.51%	99.54%	3 days, 23 h
	InParanoid	97.67%	0.89%	93.65%	99.99%	8 days, 23 h
	Proteinortho	79.36%	16.64%	97.68%	99.88%	1 h, 29 min
	PoFF	93.98%	15.52%	97.30%	99.96%	1 h, 30 min
F100	OrthoMCL/OMA/InParanoid	-	-	-	-	>31 days
	Proteinortho	23.99%	20.48%	99.37%	99.71%	6 h, 39 min
	PoFF	90.16%	18.17%	99.62%	99.99%	6 h, 44 min

Comparison of computational results with orthology relations derived from simulated datasets with different gene family sizes. Statistical values are explained in Materials and Methods. *tn* rate refers to true negative rate. Running time was measured on a quad core CPU (Intel core i7 at 2.9 GHz) with eight threads.

It would be desirable to include several other orthology detection tools to directly compare the results achieved using the simulated datasets. To our knowledge, only OrthoMCL, OMA, InParanoid and Roundup 2.0 are available as standalone tools that can be used for large input datasets. Since Roundup 2.0 largely relies on a commercial implementation of blast, we were only able to include the first two tools in the benchmark. We observed that OrthoMCL is very inclusive. It returns huge orthologous groups comprising whole gene families but, according to the results, does not reflect pairwise orthology to a reasonable extent. This results in a large number of false positive predictions. It also requires extensive computational resources: We terminated the analysis of the biggest dataset (*F*100) after 31 days of runtime without obtaining a result (using an Intel core i7 quad core CPU at 2.9 GHz). OMA and InParanoid required even more computational resources. We also had to terminate the analysis of the biggest dataset without obtaining a result from these tools. The results obtained for the two other datasets, however, were superior to those obtained from OrthoMCL. InParanoid reports the smallest amount of orthology relations (only ∼1–5% recall) and exhibits the longest runtime. The results however hardly include any false positives.

Since FFAdj-MCS acts as an efficient filter against false orthology predictions, we tested whether we could rely entirely on the synteny information. After all, this information is also derived from the alignment scores determined by blast, hence low-scoring edges are unlikely to enter the final matching and would thus be dismissed either way. We therefore removed Proteinortho's filter for near-optimal alignment scores by setting *f* = 0, which includes all reciprocal alignments above the given E-value threshold. We observed that this did not improve the quality of the predictions but increased the CPU time by a factor of 20 to 40 on the simulated datasets. A cutoff value of *f* close to 1 thus not only saves computational resources but also contributes to the identification of the correct edges in 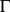 independent of FFAdj-MCS. This observation justifies the design decision to run the gene order filter only on the nearly optimal orthology candidates.

In addition to simulated data, we performed benchmarks using estimated orthology relationships from several real life datasets. The COG-database [8] was used as complete reference annotation for a set of 16 prokaryotes. All proteins present in this set are assigned to some group. OrthoBench [23] and YGOB [46] provided a partial annotation for a number of reference proteins in twelve metazoan and five fungal species, respectively. The YGOB dataset was used in a previous study to evaluate the tool Synergy [7]. While the latter is not publicly available, the results of its application to YGOB have been published, which allowed us to compare Synergy and PoFF on this dataset (see Table 3 and discussion below).

**Table 3:** Comparison using real data.

Dataset	Method	Precision	Recall	Accuracy	*tn* rate
COG	Proteinortho	99.50%	23.80%	29.12%	98.45%
	PoFF	99.52%	22.50%	27.93%	98.47%
YGOB	Synergy	61.36%	42.82%	99.64%	99.89%
	Proteinortho	59.10%	38.35%	99.62%	99.89%
	PoFF	59.07%	36.97%	99.62%	99.89%
OrthoBench	Proteinortho	100%	17.68%	24.71%	100%
	PoFF	100%	9.72%	17.44%	90.27%

Comparison of tools on the basis of estimated orthology relations from real data sets. Statistical values are explained in Materials and Methods. *tn* rate refers to true negative rate.

For real life datasets, PoFF predicts 4 to 57% fewer pairwise orthology relations than Proteinortho. This tendency is even more pronounced for the very similar simulated datasets (23 to 77%, data not shown). The reduced number of pairwise orthology relations allows separating the orthologous groups in a more fine-grained way and reduces the number of false positive assignments. In turn, however, the number of true positive assignments is reduced as well. For the real life datasets, which comprise far more distant species than the simulated data, this results in reduced recall and sometimes also reduced accuracy (Table 3).

We emphasize that neither the COG nor the OrthoBench data are ideal benchmarking sets for fine-grained orthology predictions. Both provide orthologous groups rather than pairwise orthology relations which, in turn, had to be estimated for evaluation (see Materials and Methods). Moreover, many of these groups are rather large as they contain numerous paralogs, which were – as we would argue – correctly clustered into subgroups by PoFF and/or Proteinortho. The COG-database was originally constructed using 13 Archaea, three Eukarya and 50 Bacteria. For evaluation, we used a bacterial subset of 16 species. This in turn makes duplications specific to the chosen subset harder to detect. The combination of these issues leads to artifacts in the reference datasets that might have a negative impact on recall and accuracy. Both, PoFF and Proteinortho tend to split the groups annotated in the reference sets into smaller subgroups. This effect of subdividing is more pronounced for PoFF. OrthoBench groups contain on average 23.5 genes while comprising only up to 12 species. On average these groups are divided into 3.8 subgroups by Proteinortho and 5.4 groups using PoFF. COG groups contain 18.4 genes on average. These groups are divided into 3.0 and 3.1 subgroups, respectively (see File S1).

Only the YGOB dataset offers pairwise orthology data and can thus be regarded as more exact than the other two sets. Here, the results of Proteinortho and PoFF are quite similar. Again we find the slight decrease in recall observed already for the simulated dataset. Increased phylogenetic distance decreases the positive impact on precision, which was found for the more closely related simulated datasets. The predictions for this dataset achieved by Synergy are slightly better than those of Proteinortho and PoFF. However, the algorithm relies on genome-wide reconstruction of phylogenetic gene trees and is thus far more time-consuming. Moreover, a standalone tool that applies the algorithm is currently not available.

The strategy pursued by PoFF is particularly useful to separate large orthologous groups with many co-orthologs into smaller subgroups. Typically, there is one major group for each gene family in each simulated dataset that spans all species of the original group but includes only one or a small number of genes from each species. In addition, we observe one or more “minor” groups of duplicates that contain diverged and/or largely rearranged paralogs. Using the real life dataset OrthoBench we see this trend in particular for Otoferlin, Dilute myosin heavy chain, GPS domain-containing GPCRs and S-adenosylmethionine synthetase isoform families. This type of subdivision appears useful and desirable in most practical applications of automatic orthology detectors.

The increase in runtime introduced by FFAdj-MCS is marginal for small genomes (e.g. Bacteria). For simulated data as well as the COG set we observed an increase by 1–3%. For large genomes as present in the OrthoBench set the increase was 5–10% and thus more notable. For example, the analysis of *Rattus norvegicus* and *Pan troglodytes* took 12.5h using Proteinortho and 13.5 h using PoFF applying a single thread. The memory requirements remained unchanged.

## Conclusions

Dissecting large gene families from many genomes into clusters of orthologs is not a well-posed problem. Orthology, as defined by Fitch [1], [2], is a binary relation of the set of genes. Gene duplication events typically appear in many different locations of the underlying phylogenetic tree and give rise to a complex structure of co-orthologs and paralogs at different levels. The resulting cograph nevertheless contains dense clusters that can be meaningfully associated with orthologous groups. Clustering-based orthology detection is therefore a useful pragmatic way to easily and correctly identify orthologous groups, provided duplications are absent within the phylogenetic range of the input data. It is a common feature of orthology methods, in particular those geared towards large datasets, that the orthology is approximated by a partition of the genes into groups of co-orthologs. The tool PoFF described here also follows this paradigm but provides pairwise orthology predictions in addition.

Several orthology prediction methods that avoid the explicit use of gene and species trees have been described in the literature. Most of them can be applied to large datasets only at high performance computing facilities. Their pre-computed results are usually available in databases, whereas the software itself is not available for public use or restricted in practice to small datasets. This limits their usefulness since poorly studied or newly sequenced organisms that are not (yet) available in the pre-computed results cannot easily be included in large-scale studies. PoFF is specifically designed to overcome these limitations and provides users a tool for compiling large-scale orthology datasets with moderate computational resources. Here we have shown that the combination of the fast, clustering-based orthology heuristic, Proteinortho, with the equally efficient heuristic for large-scale synteny assessment, FFAdj-MCS, leads to a substantial improvement of the data quality for related species without loss of performance. Synteny information proves to be a highly efficient filter against false-positive orthology assignments without a huge increase of the false negative rate. The extended approach, PoFF, is capable of boosting large-scale comparative studies which focus on closely related species or even strains.

**Figure 4:**
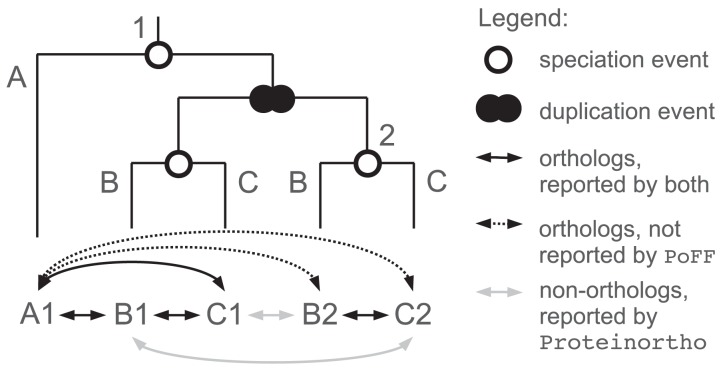
The false negative issue. The genes {*A*1, *B*1, *B*2, *C*1, *C*2} form an orthologous group. {*B*1, *B*2} as well as {*C*1, *C*2} are not orthologous to each other but co-orthologous with respect to *A*1 (*A*1 and {*B*1, *B*2, *C*1, *C*2} are separated by a speciation event). Pairwise true orthology relationships are marked by black arcs, false ones are grey. Proteinortho is more inclusive, it would report all five genes as one group, yielding six true and two false positives (grey). Assuming that the gene copies 1 and 2 exhibit distinct genomic neighborhoods in all three species *A* to *C*, PoFF would report two separate groups, namely {*A*1, *B*1, *C*1} and {*B*2, *C*2}. This more fine-grained method avoids false positive orthology assignments. However, it introduces false negative assignments. Two in this example, depicted by dashed arcs.

Orthologous groups can provide a convenient starting point for more detailed analyses of the history of entire gene families. To this end, it is necessary to reduce in particular false positive orthology assignments. Figure 4 illustrates that the filtering and clustering strategy can have a strong influence on both the false positive and false negative rates of orthology assignments. Orthology is only defined as a pairwise relationship which is not transitive. Hence, reducing the false positive rate within orthologous groups will ultimately lead to a reduction of true positive rates when the pairwise definition is applied, as we did here (see Figure 4, e.g., separating the paralogs *B*1 and *B*2 into two distinct orthologous groups requires to discard the true orthology relation to *A*1 for one of them, otherwise both genes would be connected via *A*1). Given this, we had expected PoFF to perform much worse regarding true positives, which was, however, not the case.

While conserved synteny is a powerful feature to support the confidence in orthology predictions [56], gene orders evolve faster than protein sequences [57]. This fact is reflected by the benchmark results of the closely related simulated datasets compared to the real-life sets including more distantly related species, where the advantage of PoFF regarding pairwise orthology prediction was clearly reduced (see Tables 2 and 3). However, PoFF yields orthologous groups that are more fine-grained and contain fewer paralogs. We argue that this is a practical improvement for subsequent analyses, such as gene function prediction, genome annotation, marker development and phylogenetics. There, the presence of many-to-many relations in orthologous groups due to co-orthologs may lead to inconclusive results. In turn, these groups are often omitted and single-copy orthologs (a single gene per species) are used only [58]–[60]. This fact could make an application of PoFF desirable, even for more distant species.

The extension of Proteinortho by FFAdj-MCS leads to a very moderate increase in runtime and does not increase the hardware requirements, making this combined method applicable to very large datasets further on. The current approach of combining sequence similarity, conserved synteny and clustering entails a significant improvement when comparing closely related species. As gene orders generally evolve faster than protein sequences [57], the improvement decreases with growing phylogenetic distance of species in the set, which may even compromise precision. Future extensions of the approach could thus aim at deciding on a case-by-case basis if the FFAdj-MCS algorithm should be used as additional filter for the comparison of two species, e.g., based on the respective breakpoint distance. Alternatively, a less restrictive synteny measure (e.g. common intervals instead of adjacencies) could be applied.

## Supporting Information

File S1 Table S1: Accuracy of separation of Proteinortho and PoFF evaluated in reference dataset Orthobench. Table S2: Accuracy of separation of Proteinortho and PoFF evaluated in reference dataset COG.
(PDF)
