# Supplementary material for: Orthology Detection Combining Clustering and Synteny for Very Large Datasets
Source: PLoS One. 2014 Aug 19;9(8):e105015. doi: 10.1371/journal.pone.0105015 (PMC4138177; doi:10.1371/journal.pone.0105015)
Supplement: File S1 — Table S1: Accuracy of separation of Proteinortho and PoFF evaluated in reference dataset Orthobench. Table S2: Accuracy of separation of Proteinortho and PoFF evaluated in reference dataset COG. (PDF) [file pone.0105015.s001.pdf]

S1 Supporting Information - Group composition

The following tables show how the originally defined groups in the reference datasets OrthoBench and COG are separated by Proteinortho (Po) and its extension PoFF. Members determines the number of genes in each reference group. Groups indicates into how many groups, the original reference group was divided by the respective approaches. Missing Genes gives rise to the number of original members that are not present in any group. Additional Genes tells how many members were added to the algorithm derived groups that were not present in the reference group before.

Table S1.1 OrthoBench

Group	Members	Groups		Missing Genes		Additional Genes	
		Po	PoFF	Po	PoFF	Po	PoFF
**Low Speed of Evolution**							
Split hand/Split foot 1	8	2	2	0	0	6	5
PHd Finger family	12	1	1	0	0	0	0
**High Speed of Evolution**							
Vomeromodulin	5	1	1	0	2	0	0
PLUNC proteins	60	11	15	1	3	21	17
Transient receptorchannel	41	8	11	1	2	69	36
Ionotropic glutamate receptors	7	0	0	7	7	0	0
Phophodiesterase 4D inter. pro.	11	3	5	1	1	16	16
Vitellogenin	9	3	5	2	2	11	5
LIM domain kinase	23	4	5	2	2	21	21
Otoferlin	26	4	11	0	0	19	22
**Low complexity regions/repeats**							
Sec13	11	1	4	1	1	8	8
Retinitis pigmentosa	18	4	5	4	4	5	7
Ankyrin repeat	99	15	19	3	3	41	29
Osteoclast protein	13	1	1	0	1	0	0
Dilute myosin heavy chain	32	5	9	3	3	34	38
Myosin heavy chain	41	5	10	5	6	50	48
GPS domain-containing GPCRs	50	10	16	5	5	57	42
Leucine-rich repeat	12	2	3	0	1	11	8
Laminin alpha	18	3	5	1	1	20	18
**Domain shuffling/evolution**							
Thrombospondin protein	54	9	9	4	3	15	13
Low-density lipoprotein receptors	11	1	2	1	1	2	2
FOG- Immunoglobin and related proteins	16	5	6	0	0	19	20
RNA helicase BRR2	10	1	3	0	0	4	4
**Multigene families/Paralogy**							
GPCR hormone receptors	27	4	5	0	0	18	13
Tyrosine-protein kinase	67	12	14	5	6	39	34
Cytochrome P450	27	4	5	1	2	4	6
Carbonyl reductases	24	3	5	1	1	1	2
ATP-binding cassette	31	4	8	3	4	18	15
Pyruvate dehydrogenase kinases	41	3	6	1	1	6	6
**Low Alignment Quality**							
Collagen type IV alpha chain	49	11	17	2	5	55	45
Fillagrin	6	2	2	2	2	3	4
GATA 1/2/3	32	5	4	3	6	7	7
Mucins	60	14	15	15	20	35	35
Chitinase	45	6	12	6	7	31	24
TRAF4	13	1	2	2	1	3	2
FGFR1/2/3/4	41	11	19	0	0	97	61
Serine/threonine-protein kinase	21	2	2	3	3	2	1
**High Alignment Quality**							
Ski oncogene protein	23	5	6	2	2	23	17
Ataxin-7-like protein	11	2	3	0	1	6	5
Ribosomal protein S12	13	1	4	1	0	2	2
**Random Families**							
Ribosomal protein L11	21	1	2	6	7	9	5
Methionine-R-sulfoxide reductase	18	1	3	0	1	12	11
Erythropoietin 4	15	1	1	4	4	3	3
Proline oxidase	18	3	5	1	1	18	15
Peroxisomal multifunctional enzyme	13	3	4	0	0	9	4

Ribosomal protein L37AE/L43A	24	3	4	9	9	7	5
S-adenosylmethionine synthetase isoform	26	2	4	3	3	13	7
Ribosomal protein S27E	22	2	4	2	2	10	8
Glutamate formiminotransferase	9	1	2	0	0	5	4
Alcohol dehydrogenase	12	1	2	0	0	8	5
Adenine phosphoribosyl transferase	12	2	3	1	1	4	4
Neurotensin receptor 3	10	2	2	0	0	5	4
Dystroglycan 1	11	1	2	2	2	1	1
Tyrosinase	11	1	1	1	1	2	2
C8orf13	21	3	4	3	3	4	4
Centrosomal protein	10	2	3	1	1	6	6
C1orf43	13	1	3	3	3	8	3
Tumor necrosis factor receptor superfamily	9	1	1	0	0	2	2
C6orf170	10	2	2	0	0	7	7
Calcium channel gamma subunits	74	13	13	6	7	11	11
Thyroid hormone receptor-associated protein	19	6	7	3	3	11	10
Glutathione S-transferase	11	1	1	0	0	5	5
Prokineticin 1	8	1	1	0	0	1	1
Serum response factor binding protein	13	1	1	5	5	1	1
Zinc finger, FYVE domain containing	18	4	5	4	4	5	7
C22orf25	12	2	2	0	0	5	4
Mitogen-activated protein	12	2	3	0	0	5	5
Pecanex	46	10	12	7	7	17	17
Corticotropin and Urocortin proteins	15	2	2	1	1	0	0
Histone deacetylase 3	11	1	1	0	0	3	3
**Summary**							
Sum	1642	265	377	150	174	976	802
Average/Fraction	(⌀23.5)	(⌀3.8)	(⌀5.4)	(9.1%)	(10.6%)	(39.5%)	(35.3%)
	OrthoBench	Po	PoFF	Po	PoFF	Po	PoFF
**Group**	**Members**	**Groups**		**Missing Genes**		**Additional Genes**	

Table S1.2 COG

	**Members**	**Groups**		**Missing Genes**		**Additional Genes**	
		Po	PoFF	Po	PoFF	Po	PoFF
**Summary**							
Sum	31346	5143	5344	3536	3848	3201	2901
Average/Fraction	(⌀18.4)	(⌀3.0)	(⌀3.1)	(11.3%)	(12.3%)	(10.3%)	(9.5%)
